# Genetically proxied milk consumption and risk of colorectal, bladder, breast, and prostate cancer: a two-sample Mendelian randomization study

**DOI:** 10.1186/s12916-020-01839-9

**Published:** 2020-12-02

**Authors:** Susanna C. Larsson, Amy M. Mason, Siddhartha Kar, Mathew Vithayathil, Paul Carter, John A. Baron, Karl Michaëlsson, Stephen Burgess

**Affiliations:** 1 Unit of Medical Epidemiology, Department of Surgical Sciences, Uppsala University, Uppsala, Sweden; 2 Unit of Cardiovascular and Nutritional Epidemiology, Institute of Environmental Medicine, Karolinska Institutet, 17177 Stockholm, Sweden; 3 British Heart Foundation Cardiovascular Epidemiology Unit, Department of Public Health and Primary Care, University of Cambridge, Cambridge, UK; 4 National Institute for Health Research Cambridge Biomedical Research Centre, University of Cambridge and Cambridge University Hospitals, Cambridge, UK; 5 MRC Integrative Epidemiology Unit, Bristol Medical School, University of Bristol, Bristol, UK; 6 MRC Cancer Unit, University of Cambridge, Cambridge, UK; 7 Department of Public Health and Primary Care, University of Cambridge, Cambridge, UK; 8 Department of Epidemiology, Geisel School of Medicine at Dartmouth, Hanover, NH USA; 9 Department of Medicine, University of North Carolina School of Medicine, Chapel Hill, NC USA; 10 Department of Epidemiology, Gillings School of Global Public Health, University of North Carolina, Chapel Hill, NC USA; 11 MRC Biostatistics Unit, University of Cambridge, Cambridge, UK

**Keywords:** Cancer, Genetic variants, Milk consumption, Mendelian randomization, Neoplasm

## Abstract

**Background:**

Observational studies have shown that milk consumption is inversely associated with colorectal, bladder, and breast cancer risk, but positively associated with prostate cancer. However, whether the associations reflect causality remains debatable. We investigated the potential causal associations of milk consumption with the risk of colorectal, bladder, breast, and prostate cancer using a genetic variant near the *LCT* gene as proxy for milk consumption.

**Methods:**

We obtained genetic association estimates for cancer from the UK Biobank (*n* = 367,643 women and men), FinnGen consortium (*n* = 135,638 women and men), Breast Cancer Association Consortium (*n* = 228,951 women), and Prostate Cancer Association Group to Investigate Cancer Associated Alterations in the Genome consortium (*n* = 140,254 men). Milk consumption was proxied by a genetic variant (rs4988235 or rs182549) upstream of the gene encoding lactase, which catalyzes the breakdown of lactose.

**Results:**

Genetically proxied milk consumption was associated with a reduced risk of colorectal cancer. The odds ratio (OR) for each additional milk intake increasing allele was 0.95 (95% confidence interval [CI] 0.91–0.99; *P* = 0.009). There was no overall association of genetically predicted milk consumption with bladder (OR 0.99; 95% CI 0.94–1.05; *P* = 0.836), breast (OR 1.01; 95% CI 1.00–1.02; *P* = 0.113), and prostate cancer (OR 1.01; 95% CI 0.99–1.02; *P* = 0.389), but a positive association with prostate cancer was observed in the FinnGen consortium (OR 1.07; 95% CI 1.01–1.13; *P* = 0.026).

**Conclusions:**

Our findings strengthen the evidence for a protective role of milk consumption on colorectal cancer risk. There was no or limited evidence that milk consumption affects the risk of bladder, breast, and prostate cancer.

## Background

Milk products are major components of the traditional Western diets and are rich sources of essential nutrients [1]. The association between self-reported milk consumption and cancer risk has been extensively studied. Available observational data indicate that milk consumption is associated with a reduced risk of colorectal cancer [2–7], an association that may be mediated, at least in part, by calcium [7–10]. The World Cancer Research Fund and American Institute for Cancer Research have concluded that there is convincing evidence that consumption of dairy foods and taking calcium supplements decrease the risk of colorectal cancer [7]. Inconclusive observational evidence further suggests that milk consumption is inversely associated with risk of bladder [4, 11, 12] and breast cancer [4, 7, 13, 14], positively associated with prostate cancer [4, 7, 15–17], but not associated with other cancers [4, 7, 18, 19]. However, as observational studies are susceptible to methodological biases, particularly confounding and reverse causality, the causal role of milk consumption for cancer risk remains unestablished.

Milk sugar (lactose) is digested by the enzyme lactase, which is encoded by the lactase gene (*LCT*) and produced by cells in the small intestine. A single nucleotide polymorphisms (SNP) upstream of the *LCT* gene is associated with lactase persistence (the continued activity of the lactase enzyme in adulthood) and with higher milk consumption in European populations [20, 21]. We assessed the potential causal associations of milk consumption with the risk of colorectal, bladder, breast, and prostate cancer using the *LCT* gene variant as proxy for milk consumption.

## Methods

### Outcome data sources

Genetic association estimates for colorectal, bladder, breast, and prostate cancer were estimated in the UK Biobank cohort, which enrolled about 500,000 adults (37 to 73 years of age) between 2006 and 2010 [22]. In the present analysis, we included 367,643 unrelated participants of European ancestry to diminish population stratification bias and used follow-up data to March 31, 2017. Classification of each cancer site has been reported previously [23]. Analyses of genotype-cancer associations were done only in women for breast cancer and only in men for prostate cancer. We adjusted the association estimates for age, sex (in analyses of colorectal and bladder cancer), and the first ten genetic principal components through logistic regression.

We additionally obtained summary-level data (i.e., beta coefficients and standard errors) for genetic associations with colorectal, bladder, breast, and prostate cancer from the FinnGen consortium (including up to 135,638 women and men of Finnish ancestry) [24]. The equivalent summary-level data for breast and prostate cancer were acquired respectively from the Breast Cancer Association Consortium (BCAC) (including 228,951 women of European ancestry) [25] and the Prostate Cancer Association Group to Investigate Cancer Associated Alterations in the Genome (PRACTICAL) consortium (including 140,254 men of European ancestry) [26]. All genetic association estimates were calculated by logistic regression comparing cases and controls and adjusted for genetic principal components (the first ten in the FinnGen consortium and BCAC, and the first seven in the PRACTICAL consortium). Some studies further adjusted for study-relevant covariates, such as age, sex (in analyses of colorectal and bladder cancer in the FinnGen consortium), country, and genotyping batch.

The UK Biobank and studies included in the consortia had been approved by an ethical review board, and all participants provided informed consent. The current analyses were approved by the Swedish Ethical Review Authority.

### Genetic instrument

As a genetic instrument for milk consumption in our primary analyses, we used rs4988235, which is located upstream from the *LCT* gene and is associated with milk consumption in European populations [20, 21]. In a subcohort of 12,722 participants of the European Prospective Investigation into Cancer and Nutrition-InterAct study, the median milk consumption was 162 g/day (25th to 75th percentile, 37 to 300 g/day) and each additional milk intake increasing allele of rs4988235 was associated with an increase in milk consumption of 17.1 g/day (*P* = 2 × 10^−7^) [20]. In a population-based cohort study of 73,715 Danish individuals, milk consumption increased with 0.58 (95% CI 0.49–0.68) glasses/week (*P* = 9 × 10^−36^) for each additional milk intake increasing allele of rs4988235 [21]. In the Danish cohort, the SNP explained 2% of the variance in milk intake, and the F-statistic was 515 [21]. Rs4988235 was not available in the FinnGen consortium, and a proxy SNP (rs182549) in complete linkage disequilibrium was used.

Another SNP (rs3754686) nearby the *LCT* gene has been shown to be strongly associated with milk consumption in Mediterranean and American populations [27]. Rs3754686 was available in all data sources and was in strong linkage disequilibrium with rs4988235 (*R*^2^ = 0.71 in the CEU [Utah residents from North and West Europe] population, 0.95 in the British population, and 0.77 in the Finnish population; LDlink version 4.2 [28]). Rs3754686 was used as an instrument for milk consumption in a complementary analysis.

### Statistical analysis

Odds ratio (OR) estimates were reported per additional milk intake increasing allele. Estimates from the different data sources were combined using fixed-effects meta-analysis. The amount of heterogeneity between estimates was quantified using the *I*^2^ statistic [29]. All analyses were conducted using Stata/SE 14.2 (Stata Corporation, College Station, TX, USA).

## Results

**Fig. 1:**
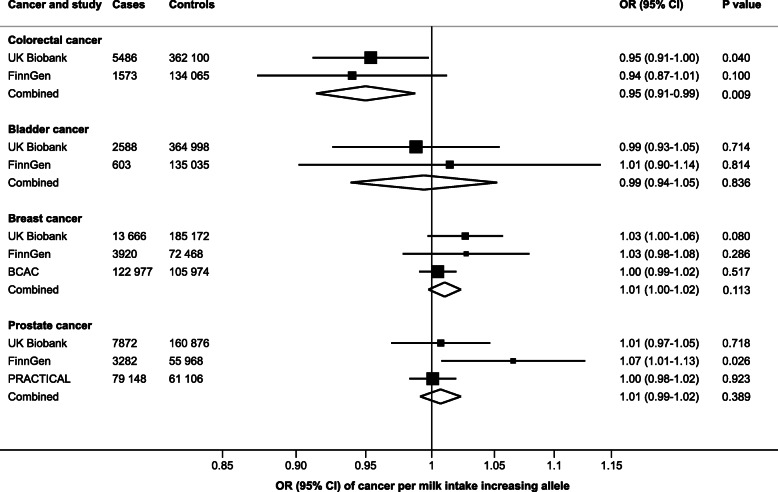
Associations of higher milk consumption with colorectal, bladder, breast, and prostate cancer. Estimates are per milk intake increasing allele of rs4988235. In the FinnGen consortium, a proxy variant (rs182549) in complete linkage disequilibrium with rs4988235 was used. *BCAC* Breast Cancer Association Consortium, *CI* confidence interval, *OR* odds ratio, *PRACTICAL* Prostate Cancer Association Group to Investigate Cancer Associated Alterations in the Genome consortium

Genetically predicted milk consumption was inversely associated with colorectal cancer risk in the combined analysis of the UK Biobank and FinnGen consortium (Fig. 1). The OR for each additional milk intake increasing allele was 0.95 (95% confidence interval [CI] 0.91–0.99; *P* = 0.009), without evidence of heterogeneity between estimates from the two studies (*I*^2^ = 0%). Genetically predicted milk consumption was not associated with bladder cancer (OR 0.99; 95% CI 0.94–1.05; *P* = 0.836; *I*^2^ = 0%) or breast cancer (OR 1.01; 95% CI 1.00–1.02; *P* = 0.113; *I*^2^ = 5.4%) (Fig. 1). In the BCAC, results were null for both estrogen receptor-positive (OR 1.00; 0.98–1.02; *P* = 0.999) and estrogen receptor-negative (OR 1.01; 95% CI 0.98–1.04; *P* = 0.494) breast tumors. No overall association was observed between genetically predicted milk consumption and prostate cancer (OR 1.01; 95% CI 0.99–1.02; *P* = 0.389), but there was moderate heterogeneity among estimates from different data sources (*I*^2^ = 54%) and a positive association was observed in the FinnGen consortium (OR 1.07; 95% CI 1.01–1.13; *P* = 0.026) (Fig. 1).

Results were similar when using rs3754686 as an instrument for milk consumption. The ORs in these analyses for each additional milk intake increasing allele were 0.94 (95% CI 0.90–0.90; *P* = 0.003) for colorectal cancer, 1.01 (95% CI 0.95–1.07; *P* = 0.767) for bladder cancer, 1.00 (95% CI 0.99–1.01; *P* = 0.974) for breast cancer, and 1.01 (95% CI 0.99–1.03; *P* = 0.265) for prostate cancer.

## Discussion

This genetic study found that higher milk consumption was associated with a reduced risk of colorectal cancer, consistent with findings of observational studies [2–7]. However, our findings do not support observational findings that higher milk consumption is associated with a lower risk of bladder and breast cancer or with a higher risk of prostate cancer, though a positive association between genetically predicted milk consumption and prostate cancer was found in the FinnGen consortium. For bladder cancer, observational studies have reported a protective association mainly with the consumption of fermented milk products (cultured milk, yogurt, and cheese) [12, 30]. The lactase persistence variant is not associated with yogurt and cheese consumption [20, 21].

Studies of genetically predicted milk consumption in relation to site-specific cancers are scarce. No association was observed between rs4988235 and colorectal cancer in small case-control studies conducted in Turkey (44 cases and 48 controls) [31] and Italy (306 cases and 311 controls) [32]. Lactase persistence was non-significantly positively associated with prostate cancer risk in a meta-analysis of three studies, including a case-control study nested in the European Prospective Investigation into Cancer and Nutrition study (630 cases and 873 controls) and case-control studies of Finnish (1229 cases and 473 controls) and Swedish (2924 cases and 1842 controls) men [33]. The combined OR was 1.12 (95% CI 0.96–1.32) [33] and ranged from 1.06 to 1.16 in individual studies [33, 34]. This finding is consistent with the result observed in the FinnGen consortium. The reason for the disparate findings for rs4988235 and prostate cancer risk might be related to different amounts of milk consumed in different populations. Finland is the country with the highest per capita milk consumption and Sweden also has high consumption [35]. If there is a threshold effect of milk consumption on prostate cancer risk, an association between the lactase persistence variant and prostate cancer might only be seen in populations with high milk consumption.

The observed protective association between milk consumption and colorectal cancer may be mediated, at least partly, by calcium. Calcium supplementation has been demonstrated to reduce the risk of colorectal adenomas in randomized controlled trials [8] and colorectal cancer in observational studies [9, 10]. In one of the trials, the protective effect of calcium supplementation on colorectal adenomas was confined to individuals with a normal body mass index [36]. Calcium may have a local protective effect on colorectal cancer by binding secondary bile acids and free fatty acids in the lumen of the bowel, thereby inhibiting their toxic effects on colonocytes and suppressing mucosal proliferation [37, 38]. Stimulation of the calcium-sensing receptor in colonocytes is another possible mechanism [39]. Calcium supplementation has also been shown to lead to a non-significant increased expression of tight junction proteins, suggesting that calcium may play a role in maintaining the integrity of the intestinal mucosal barrier [40].

Other compounds in milk that might protect against colorectal cancer include butyric acid (a short-chain fatty acid), conjugated linoleic acid, sphingolipids, and lactoferrin [37, 38, 41]. Almost 70% of the fat in milk is saturated of which about 11% comprises short-chain fatty acids (half of which is butyric acid) [42]. Milk fat also contains conjugated linoleic acid [42]. The anti-carcinogenic effects of this fatty acid have been demonstrated in animal models [37], but human data are limited to a Swedish cohort study which revealed a statistically significant inverse association between conjugated linoleic acid intake and risk of colorectal cancer [43]. Lactoferrin is an iron-binding glycoprotein present in human and bovine milk. It enhances immune function and inhibits colorectal carcinogenesis in animal models [44]. In addition, a randomized controlled trial found that orally administered lactoferrin for 12 months retarded the growth of adenomatous colorectal polyps in the subgroup of participants 63 years of age or younger [44]. Milk consumption may also influence colorectal cancer risk by altering the gut microbiota. Higher milk consumption has been associated with greater microbiota richness and more abundant *Faecalibacterium* and *Fusobacterium* but less *Bacteroides* in humans [45]. A recent study showed that supplementing the diet of aging mice with bovine milk increased intestinal levels of short-chain fatty acids through modulation of gut microbiota [46].

There is suggestive or weak evidence from observational studies that milk consumption might increase prostate cancer risk via calcium [7, 17] and insulin-like growth factor 1 [47]. A recent Mendelian randomization study showed that genetically predicted insulin-like growth factor 1 levels were significantly positively associated with prostate cancer in the UK Biobank and non-significantly positively associated with prostate cancer in the PRACTICAL consortium and BioBank Japan [48]. Milk is also a rich source of phosphorus, which was observed to be positively associated with the risk of prostate cancer in a cohort of US health professionals [49].

Findings from a Mendelian randomization study can be biased if the genetic instrument affects the outcome through a pathway other than via the exposure of interest. In this case, it is plausible that a high milk consumption results in lower or higher consumption of other foods. In fact, the milk intake increasing allele of rs4988235 has been shown to be modestly associated with lower consumption of fruits (− 7.0 g/day, *P* = 0.01), cereals (− 3.4 g/day, *P* = 0.03), poultry (− 0.8 g/day, *P* = 6 × 10^−3^), and wine (− 4.8 g/day, *P* = 0.03) but with higher consumption of potatoes (3.0 g/day, *P* = 5 × 10^−3^) in European individuals [20]. An association between the milk intake increasing allele of rs4988235 and lower consumption of fruits as well as vegetables was also observed in a Danish population [50]. There is some evidence that low consumption of fruits and vegetables might increase the risk of colorectal cancer [5, 7]. Thus, any effect of fruit and vegetable consumption would be expected to attenuate the association between genetically predicted milk consumption and risk of colorectal cancer towards the null and cannot explain our finding.

The milk intake increasing allele of rs4988235 has also been associated with higher body mass index in several studies [51–53] but not all [20, 21], as well as positively associated with height [54]. Considering that greater body mass index and height are associated with an increased risk of colorectal cancer [55, 56], these factors also cannot mediate the inverse association between genetically predicted milk consumption and colorectal cancer. Rs4988235 is not associated with other potential colorectal cancer risk factors, such as red meat, processed meat, and alcohol consumption, type 2 diabetes, physical activity, and smoking, and is also not associated with education level in European individuals [20, 21, 50].

Variations in allele frequency of the lactase persistence variant across populations can lead to population stratification bias. In this MR study, we reduced such bias by confining the analyses to individuals of European ancestry. Furthermore, all studies adjusted for population substructure through genetic principal components. Results for genetically predicted milk consumption and colorectal cancer risk were also consistent in the relatively homogenous study sample of Finnish participants included in the FinnGen consortium, suggesting that population stratification bias is unlikely to explain this association. A possible explanation for the observed positive association between genetically predicted milk consumption and prostate cancer risk in FinnGen might be related to that the FinnGen population is more homogenous than the populations in the other two data sources.

## Conclusions

Our findings strengthen the evidence for a protective role of milk consumption on colorectal cancer risk. There was no or limited evidence that milk consumption affects the risk of bladder, breast, and prostate cancer.

## Data Availability

Information on how to obtain summary-level data from the FinnGen consortium, the BCAC, and the PRACTICAL consortium is available at https://finngen.gitbook.io/documentation/, http://bcac.ccge.medschl.cam.ac.uk/, and http://practical.icr.ac.uk/blog/, respectively. Access to the UK Biobank data can be obtained upon application (https://www.ukbiobank.ac.uk/). Summary-level data analyzed in this study can be obtained from the corresponding author upon reasonable request.

## Abbreviations

BCAC: Breast Cancer Association Consortium
CI: Confidence interval
OR: Odds ratio
PRACTICAL: Prostate Cancer Association Group to Investigate Cancer Associated Alterations in the Genome
SNP: Single nucleotide polymorphisms
